# Scale-Up of Integrated Care Interventions for Chronic Diseases in Diverse Settings

**DOI:** 10.5334/ijic.8981

**Published:** 2024-09-10

**Authors:** Grace Marie Ku, Zalika Klemenc-Ketiš, Antonija Poplas-Susič, Roy Remmen, Wim Van Damme, Edwin Wouters, Josefien Van Olmen, Kerstin Klipstein-Grobusch

**Affiliations:** 1 Institute of Tropical Medicine, Antwerp, Belgium; 2 Vrije Universiteit Brussel, Brusssel, Belgium; 3 University of Santo Tomas, Manila, Philippines; 4 Community Health Centre Ljubljana, Ljubljana, Slovenia; 5 Department of Family Medicine, Medical Faculty, University of Maribor, Maribor, Slovenia; 6 Department of Family Medicine, Medical Faculty, University of Ljubljana, Ljubljana, Slovenia; 7 Community Health Centre Ljubljana, Ljubljana, Slovenia; 8 University of Antwerp, Antwerp, Belgium; 9 School of Public Health, University of the Witwatersrand, Johannesburg, South Africa; 10 Julius Center for Health Sciences and Primary Care, University Medical Center Utrecht, Utrecht University, Utrecht, The Netherlands

**Keywords:** integrated care, diabetes mellitus type 2, hypertension, scaling-up

## Background

The global burden of chronic conditions such as Type 2 Diabetes (T2DM) and hypertension (HTN) is increasing exponentially, not only in high-income countries (HIC) but also, and more rapidly, in low- and middle-income countries (LMIC). Health systems worldwide struggle to manage this growing burden. Integrated interventions for prevention, detection, treatment, and control of T2DM and HTN have been previously demonstrated to be effective in controlled or small-scale settings [1,2]. However, complex organisational approaches are needed to implement such interventions nationwide in a large-scale manner. These approaches require system changes and coordinated actions from many different actors and institutions to produce maximal effect.

Scaling-up has proven to be challenging all around the world, producing mixed outcomes in both HICs and LMICs. Better understanding of how to address the systemic issues of scale-up is needed to develop and document strategies for sustainable and effective scale-up of such interventions. Global challenges and health system constraints urge us to think and work across contexts. Through reciprocal learning between different contexts, we can increase the understanding of the relationships between context, intervention, implementation and impact [3] for broad application of sustainable and effective scale-up models in different settings.

### Scale-up Integrated care for Diabetes and hypertension (SCUBY) project

The SCUBY project (see **Box**) examined how to scale-up an existing evidence-based Integrated Care Package (ICP) for control of T2DM and/or HTN. The ICP consists of five components: (a) identification of people with HTN and/or T2DM, (b) treatment in primary care services, (c) health education, (d) self-management support to patients and caregivers, and (e) collaboration among caregivers. The ICP and its interventions are in line with models for chronic care, WHO guidelines on integrated care, and essential interventions for diabetes and hypertension [4,5,6,7]. Furthermore, there is strong evidence that this ICP, when successfully implemented, leads to improved care processes and responsiveness of health care to patients’ needs and to better health outcomes [8].

**Box 1 The SCUBY Project**

SCUBY or ‘Scaling up an integrated care package for diabetes and hypertension for vulnerable people in Cambodia, Belgium & Slovenia (SCUBY)’ was carried out between 2019 and 2023 by five partners: the National Institute of Public Health (Cambodia); the Community Health Centre Ljubljana (Slovenia); the University Medical Center Utrecht (The Netherlands); and the University of Antwerp and the Institute of Tropical Medicine, Antwerp (Belgium). The project implementation sites were Cambodia, Slovenia, and Belgium (through the University of Antwerp). The Institute of Tropical Medicine coordinated the project, and the University Medical Center Utrecht led the process and impact evaluations.

Each implementation country chose to concentrate on one or more axes of scale-up [9] of the integrated care package: increase in population coverage; expansion of the ICP; and institutionalization of the ICP into the health system, referring to the degree to which governance arrangements, organization, financing and service delivery allow the ICP to be adopted and diffused in the health system [8]. The study design is described in an earlier paper [10].

Descriptions of some per-country pilot implementation [11,12] and process evaluation of the project [13] have also been presented elsewhere.

*SCUBY was funded by the European Union’s Horizon 2020 research and innovation programme under grant agreement No 825432. Views and opinions expressed are those of the author(s) only and do not necessarily reflect those of the European Union. Neither the European Union nor the granting authority can be held responsible for them*.

Country-specific scale-up strategies and roadmaps were co-developed by the SCUBY partners with a wide range of stakeholders including policy makers, health care professionals, community groups, and patients (organizations) in three purposively selected countries representing different types of health systems: a low-middle income country with a developing health system (Cambodia), a Central European country with a centralised health system (Slovenia), and a Western European country with a decentralised system (Belgium).

## The IJIC Special Issue

This Special Collection aims to provide guidance on scale-up of integrated care interventions for chronic diseases in varied contexts based on evidence from the SCUBY experience, covering three domains.

1. ***Meaning, gaps and contemporary approaches to scaled-up integrated chronic disease interventions***.
  We noted a conceptual lack of agreement due to a lack of standardized terms and addressed this with the development of a generic codebook for qualitative research on integrated care implementation for hypertension and diabetes, which facilitates cross-country assessments across micro-, meso-, and macro-levels [14]. The current practice of process evaluations of scale-up of complex interventions like the ICP was assessed through a scoping review, and process evaluation as a recurrent continuous activity was recommended [15].
2. ***Processes and challenges for scale-up of integrated care interventions*** for chronic diseases.
  We evaluated the level of ICP implementation and links with health systems characteristics [16]. We demonstrated how a shortage of resources, a pluriform health service landscape, and the (lack of) standardized approach influence implementation. Similar ICP implementation evaluations were conducted per country [17,18].
  Expanding on the preceding, an analysis of the health system barriers and facilitators for scale-up of the ICP was done in a multi-country case study [19]. We found that the nature of barriers and facilitators was similar and consistent across thre three settings, although the relative importance of the barriers are different. Results complement country-specific research on the topic, which we have published elsewhere [20,21,22,23].
  We also utilised Cascades of Care (CoC) analysis and mapped out the CoC for hypertension in Cambodia, Slovenia and Belgium to analyse differences between countries [24] taking into account the level of ICP implementation [15] and health system barriers [19]. In all three countries, less than 50% of people diagnosed with hypertension were well-controlled, but there were substantial differences between the three countries as to when and how many people are lost along the continuum of care. Per-country CoC analyses were reported previously for Cambodia and Belgium [25,26].
  From the users‘ perspective, we explored how individuals living with complications of T2DM experience health care in Belgium [27]. This study illustrates that even in a well-resourced health system, optimal and continuous care is challenging for subgroups of patients due to a mismatch between the professionals and system design, and the clinical and psychosocial needs of these people.
3. ***Outputs, outcomes and impact*** of the ***scale-up intervention of SCUBY***.
  Relative to the scale-up of the ICP, we conducted a costing study to look into the cost of scaling-up of an ideal minimum integrated care package in a low resource setting, Cambodia [28]. The feasibility of using digital technology to enhance integrated care was assessed by piloting telehealth to support self-management of vulnerable populations with T2DM and HTN in Slovenia [29]. Finally, we present SCUBY’s main deliverable, the different roadmaps for scaling-up integrated care for Cambodia, Slovenia, and Belgium [30]. We analysed the scale-up roadmaps across the three countries and showed how the country roadmaps reflect the differences in the historical context and current realities between the three distinct health systems. We also concluded that the roadmaps are partly shaped by the expertise and proximity to power of the country-specific change groups developing them.

## Lessons (to be) learnt

Scaling-up integrated care can have common elements across countries, as demonstrated through the three country roadmaps we co-developed: 1) creating an enabling environment for ICP implementation; 2) task-shifting to decentralise and involve patients and carers; and 3) strengthening monitoring and evaluation of scaling-up. These elements could also be useful in other settings with similar contexts, and are likely to be relevant in other countries as well. However, it should be noted that the multiple components and stakeholders interact in unique ways in each of the implementation country, with emergent effects and changes over time.

## Conclusion and Recommendations

Scale-up research is evolving as a subdomain of implementation research, with emerging paradigms. The SCUBY scale-up study explicitly took a complexity perspective on scale-up as adaptive change in a system emphasizing unpredictability, self-organization, and interdependencies. Our findings show the feasibility of using this perspective.

Countries can accelerate the scale-up of ICP for chronic diseases through national policies with guidelines for nationwide rollout, while allowing local implementers the decision-space for local adaptation.

Additional requisites may include:

- Securing appropriate and adequate support for scale-up of ICPs, including funding
- Multi-sectoral collaboration between government, healthcare providers, and community organizations
- Task redistribution and use of digital technologies
- Training programs for healthcare professionals to supplement their skills in delivering integrated care across the continuum of care.
- Increased community engagement and patient participation to ensure care packages are person-centered and culturally appropriate.
- Continuous monitoring and quality improvement mechanisms to refine and enhance ICPs based on feedback and outcomes.
